# Three-dimensional acetabular orientation measurement in a reliable coordinate system among one hundred Chinese

**DOI:** 10.1371/journal.pone.0172297

**Published:** 2017-02-16

**Authors:** Henghui Zhang, Yiping Wang, Songtao Ai, Xiaojun Chen, Liao Wang, Kerong Dai

**Affiliations:** 1 Shanghai Key Laboratory of Orthopaedic Implants, Department of Orthopaedics, Shanghai Ninth People's Hospital, Shanghai Jiao Tong University School of Medicine, Shanghai, China; 2 Institute of Biomedical Manufacturing and Life Quality Engineering, State Key Laboratory of Mechanical System and Vibration, School of Mechanical Engineering, Shanghai Jiao Tong University, Shanghai, China; 3 Department of Radiology, Shanghai Ninth People's Hospital, Shanghai Jiao Tong University School of Medicine, Shanghai, China; Louisiana State University, UNITED STATES

## Abstract

Determining three-dimensional (3D) acetabular orientation is important for several orthopaedic scenarios, but the complex geometries of both pelvis and acetabulum make measurements of orientation unreliable. Acetabular orientation may also differ between the sexes or racial groups. We aimed to (1) establish and evaluate a novel method for measuring 3D acetabular orientation, (2) apply this new method to a large population of Chinese subjects, and (3) report relevant characteristics of native acetabular orientation in this population. We obtained computed tomography scans taken for non-orthopaedic indications in 100 Chinese subjects (50 male, 50 female). A novel algorithm tailored to segmentation of the hip joint was used to construct 3D pelvic models from these scans. We developed a surface-based method to establish a reliable 3D pelvic coordinate system and software to semi-automatically measure 3D acetabular orientation. Differences in various acetabular orientations were compared within and between subjects, between male and female subjects, and between our subjects and subjects previously reported by another group. The reported method was reliable (intraclass correlation coefficient >0.999). Acetabular orientations were symmetrical within subjects, but ranged widely between subjects. The sexes differed significantly in acetabular anteversion (average difference, 3.0°; *p* < 0.001) and inclination (1.5°; *p* < 0.03). Acetabular anteversion and inclination were substantially smaller among our Chinese subjects than previously reported for American subjects. Thus, our method was reliable and sensitive, and we detected sex differences in 3D acetabular orientation. Awareness of differences between the sexes and races is the first step towards better reconstruction of the hip joint for all individuals and could also be applied to other orthopaedic scenarios.

## Introduction

The orientation of the acetabular component in total hip arthroplasty (THA) is generally described by its inclination and anteversion angles, which traditionally have three definitions: anatomical, radiographic, and operative orientations [1]. However, few studies have examined the orientation of native acetabulum with these three definitions [2, 3]. Understanding native acetabular orientation is important in orthopaedic scenarios, including THA, periacetabular osteotomies, differential diagnosis of hip osteoarthritis, and correction of femoroacetabular impingement [4–6].

Traditionally, acetabular orientation was assessed directly on cadavers or in living subjects by radiographic measurement on X-ray film or axial computed tomography (CT) images [3, 7]. Due to the complexity of pelvic anatomy and inconsistency of pelvic posture during the generation of radiographs or CT images, it is well established that these methods do not produce accurate assessments of acetabular orientation [2, 8]. With the advancement of image processing technologies, a three-dimensional (3D) pelvic model can be reconstructed based on axial CT images, which improves the reliability of the acetabular orientation measurement by taking pelvic posture into consideration. A best-fit plane or circle has been applied to indicate the 3D orientation of the acetabulum [9–12]. However, manual acquisition of anatomical landmarks is generally a time-consuming and error-prone procedure. Meanwhile, although 3D acetabular orientation has been examined [4], no study has examined and described relevant characteristics of 3D acetabular orientation among normal Chinese subjects.

We hypothesized that a reliable 3D pelvic coordinate system would provide a consistent 3D measurement of acetabular orientation, and that significant differences of 3D acetabular orientation exist between the sexes. Thus, the goals of this study were (1) to develop a new method for the measurement of 3D acetabular orientation according to the established definitions of acetabular inclination and anteversion by semi-automatically identifying pelvic anatomical landmarks and acetabular rim points, and to statistically quantify the reliability of the reported method, (2) to measure 3D acetabular orientation in a large population of Chinese subjects, and (3) to report relevant characteristics of acetabular orientation of Chinese subjects with normal pelvic anatomy, including differences between the sexes.

## Materials and methods

### Materials

After receiving approval from our institutional review board, we identified 100 sets of high-resolution CT angiography scans of the lower limbs, including the whole pelvis, from our institution’s database; these scans had been obtained for non-orthopaedic indications. For this type of study, formal consent is not required. The only inclusion criterion was age 18 to 60 years. CT scans from patients referred for hip pain or from individuals with apparent evidence of dysplasia, osteoarthritis, fracture, tumors, or any previous surgery around the hip were excluded.

CT images were acquired using Siemens SOMATOM Definition Flash 128 scanners at a slice thickness of 1 mm and an average in-plane (x-y) resolution of 0.98 pixel. The cohort consisted of 50 males and 50 females with a mean age of 46.9 years (range, 18 to 60 years). The DICOM (Digital Imaging and Communications in Medicine) standardized image files for each subject were exported for further analysis.

### Data extraction

**Fig 1:**
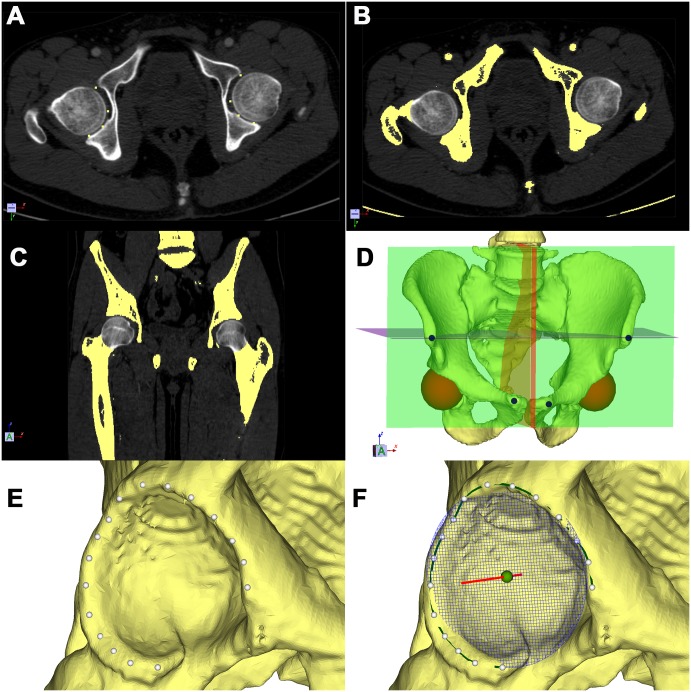
Schematic showing the segmentation, modeling, and 3D measurement of acetabular orientation. **(A), (B), (C)** Points (yellow) were manually located at the joint space on axial and coronal sections of CT images to isolate the femoral head from the pelvis in **(A)**, and then expanded computationally to produce the yellow areas shown in **(B)** and **(C)**. Spherical masks (gray) are shown on the transverse and coronal planes. **(D)** A red spherical mask was created by a novel algorithm. A virtual pelvic model (yellow) was reconstructed using threshold and region-growing algorithms. Four initial landmarks were manually located on the bilateral anterior superior iliac spines and pubic tubercles (dots). **(E)** About 20 points (white) were manually located on the acetabular rim. **(F)** A B-spline path (green) was built as the rim path using cubic interpolation. The acetabular opening circle and axis were created subsequently. A best-fit circle (blue mesh) was created with a least-squares method. The center of rotation (green sphere) and the axis perpendicular to the opening plane (red line) were computed.

Using the 3D Acetabulometer software developed in-house by our engineers (Y.P.W., X.J.C.), 3D pelvic models were virtually reconstructed from the DICOM data. In order to maintain the natural surface contours, each model was minimally smoothed and without surface simplifications. A few specific data elements were manually extracted for each subject in 3D Acetabulometer. A spherical mask was manually fitted to remove the femoral head from CT volume images (Fig 1). After the femoral head was isolated from the pelvis, the pelvis was easily segmented using a region-growing algorithm. Finally, four initial bony landmarks, the bilateral anterior superior iliac spines (ASISs) and pubic tubercles (PTs), were manually identified on the reconstructed pelvic models to begin the analysis (Fig 1).

### Computational analysis

**Fig 2:**
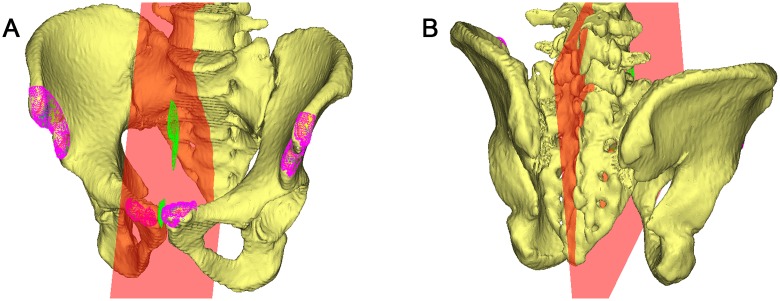
Establishment of a reliable coordinate system. By manually identifying anterior superior iliac spines and pubic tubercles, a larger area defined as a point cloud (pink) was automatically selected, and the midsagittal pelvic plane (red) was computed from these points using an iterative closest-point algorithm.

**Fig 3:**
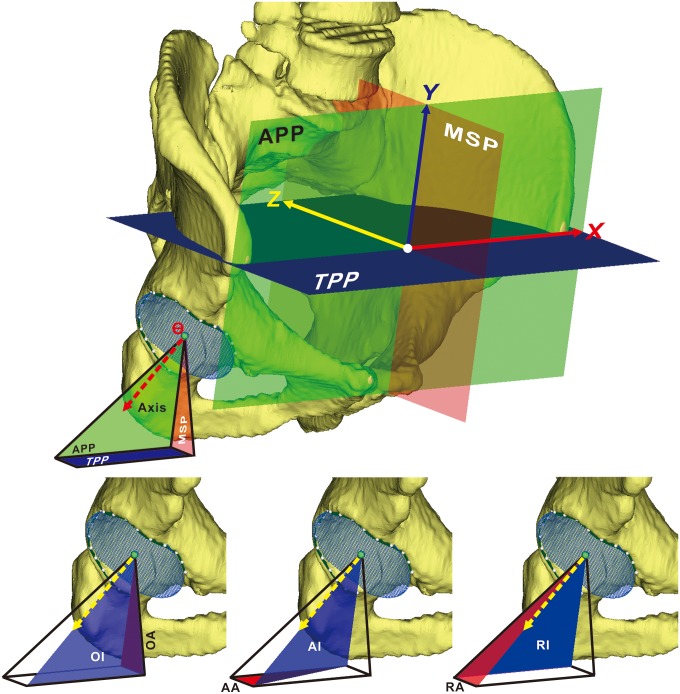
Schematic showing the pelvic coordinate system and the angular definitions of acetabular orientation. **(Top)** The anterior pelvic plane (APP; green) is determined by the bilateral anterior superior iliac spines and the midpoint of the pubic tubercles. The midsagittal plane (MSP; red) is the mirror plane determined by both ASIS regions using an iterative closest-point algorithm. The transverse pelvic plane (TPP; blue), is a plane perpendicular to both APP and MSP, at the level of the ASISs. **(Bottom)** Standard measures of anteversion (red) and inclination (blue) of the acetabular axis (yellow arrow) on the right acetabulum, showing operative anteversion (OA) and inclination (OI) (left), anatomic anteversion (AA) and inclination (AI) (middle), and radiographic anteversion (RA) and inclination (RI) (right).

To measure acetabular orientation in 3D, we established a reliable 3D pelvic coordinate system by determining the anterior pelvic plane (APP), mid-sagittal plane (MSP), and transverse pelvic plane (TPP) (Fig 1). By manually selecting bilateral ASISs and PTs on the surface of pelvic models, a plane was created virtually, providing initial estimates of the APP. To minimize the inherent uncertainty in the APP caused by manual selection, a unique iterative algorithm was developed to automatically determine the most ventral aspect of both ASISs and the midpoint of the PTs. The orientation of APP was iteratively improved until consecutive iterations produced the same points, indicating that the algorithm had converged on the most anterior points of the APP. The MSP was computed as the mirror plane associated with both ASIS regions by using an iterative closest-point algorithm (Fig 2). Then the TPP was determined as the plane perpendicular to both APP and MSP (Fig 3). The pelvic posture was assessed for each individual by measuring the pelvic tilt, rotation, and obliqueness [13]. Pelvic tilt was defined as the angle between the APP and the coronal plane, pelvic rotation was defined as the angle between the MSP and the sagittal plane, and pelvic obliqueness was defined as the angle between the TPP and the axial plane.

After the pelvic coordinate system was established, about 20 points evenly distributed on the acetabular rim, excluding the acetabular notch, were manually selected to generate a best-fit circle representing the acetabular opening surface by using a least-squares method. The acetabular axis was defined as the axis perpendicular to the best-fit circle of the opening surface that passed through the center of the circle, representing the actual 3D acetabular orientation [11, 14]. The orientation of the acetabular axis was then converted to anatomic, radiographic, and operative angular definitions of inclination and anteversion based on the standardized pelvic coordinate system, describing the orientation of the acetabulum in 3D space (Fig 3).

### Statistical analysis

**Fig 4:**
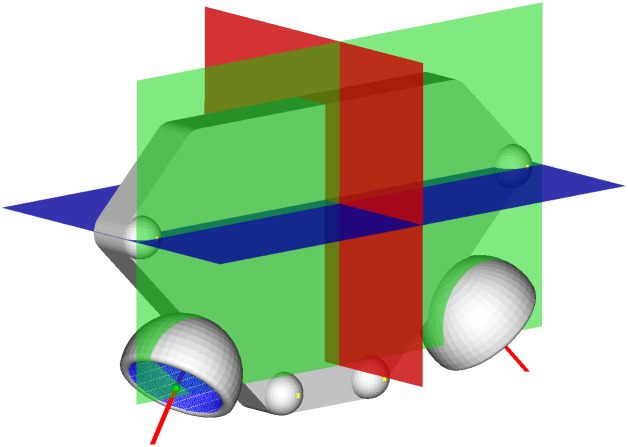
Systematic error evaluation. Standardized, simplified 3D virtual pelvic models were created using SOLIDWORKS^™^ 2015 software (Dassault Systemes, Waltham, Massachusetts) and imported into 3D Acetabulometer as the true (predetermined) value of the 3D orientation, for comparison with the value measured with the algorithm.

To assess the systematic errors caused by the algorithm applied in 3D Acetabulometer, 15 standardized 3D pelvic models were created using SOLIDWORKS^™^ 2015 software (Dassault Systemes, Waltham, Massachusetts, USA). The models were based on the anatomic parameters predetermined from the CT scans, including the coordinates of bilateral ASISs, PTs, the centroids of best-fit circles, and 3D angular data of acetabula (Fig 4). The simplified pelvic models were then imported into 3D Acetabulometer, and the anatomic, radiographic, and operative inclination and anteversion were measured. The systematic errors of the 3D acetabular orientation measured by 3D Acetabulometer were calculated by comparing the differences between the predetermined and measured 3D acetabular orientation.

The intraclass correlation coefficient (ICC) was calculated to evaluate the reliability of the reported method, and a group of typical users (H.H.Z., Y.P.W., and L.W.) were enrolled to analyze the interobserver and intraobserver ICCs. To accommodate an estimated ICC of 0.9 and a desired 95% confidence interval (95% CI; α = 0.05) with a width of 0.2° [12, 15], we randomly selected 16 subjects (32 hips) from the main data set by using a module of GraphPad Prism^™^ version 6.00 (GraphPad Software, La Jolla, California, USA) to generate random numbers. Raters started with DICOM images and performed all operations such as thresholding, segmentation, reconstruction, and initial bony landmark identification using 3D Acetabulometer. Three trials were independently performed about two weeks apart by the three raters on all 32 hips.

To calculate the interobserver ICC, a two-way analysis of variance (ANOVA) model was applied to explain random effects of different raters and of study subjects [15, 16]. To calculate the intraobserver ICC [16], a two-way mixed-effects ANOVA model was used, which treated the raters as fixed and the subjects as random effects. Both the intraobserver and interobserver ICCs were calculated to describe the reliability of the measurement of inclination and anteversion grouped within the established angular definitions (operative, radiographic, and anatomic).

The inclination and anteversion were compared between males (n = 100 hips) and females (n = 100 hips) using unpaired t-tests within each angular definition. The difference between the orientation of bilateral acetabula was also examined, using paired two-sample t-tests.

To compare our results with those from the study of Higgins et al. [4], we subtracted our mean values from their mean values for the overall groups, males only, and females only for each acetabular orientation (anteversion and inclination for the anatomic, radiologic, and operative definitions). Unpaired t-tests were used to determine the statistical significance of the differences. Differences were considered significant when *p* was <0.05.

## Results

When we compared the differences between the standardized models based on predetermined parameters and the computationally measured 3D acetabular orientation, 3D Acetabulometer achieved high accuracy, with a mean error of 0.203° (range 0.027° to 0.656°). Near-perfect reliability was found for both the interobserver and intraobserver ICC analyses (Tables 1 and 2) for all three measurement schemes.

**Table 1:** Single-measure interobserver reliability.

Measure	Anatomic	Radiographic	Operative
Trial 1	0.9997 (0.9994 to 0.9999)	0.9998 (0.9995 to 0.9999)	0.9995 (0.9992 to 0.9999)
Trial 2	0.9999 (0.9998 to 0.9999)	0.9998 (0.9997 to 0.9999)	0.9998 (0.9992 to 0.9999)
Trial 3	0.9998 (0.9996 to 0.9999)	0.9998 (0.9996 to 0.9999)	0.9997 (0.9996 to 0.9998)

The interobserver intraclass correlation coefficient (ICC) scores are given, with the 95% confidence interval in parentheses, for single measures in terms of absolute agreement (an ICC of approximately 0.90 to 1.00 for Cronbach alpha can be considered almost perfect).

**Table 2:** Single-measure intraobserver reliability.

Rater	Anatomic	Radiographic	Operative
H.H.Z.	0.9998 (0.9996 to 0.9999)	0.9998 (0.9996 to 0.9999)	0.9989 (0.9976 to 0.9997)
Y.P.W.	0.9910 (0.9979 to 0.9996)	0.9990 (0.9987 to 0.9996)	0.9924 (0.9826 to 0.9971)
W.L.	0.9998 (0.9997 to 0.9999)	0.9997 (0.9996 to 0.9999)	0.9986 (0.9974 to 0.9994)

The intraobserver ICC scores are given, with the 95% confidence interval in parentheses, for single measures in terms of absolute agreement.

The mean pelvic tilt was 2.64° (–12.65° to 19.21°), the mean pelvic rotation was 3.35° (0.34° to 13.19°), and the mean pelvic obliqueness was 5.91° (0.48° to 23.37°) (Table 3). No significant differences were found between males and females in pelvic tilt, rotation, or obliqueness (Table 3). Acetabula were significantly more anteverted in females than in males (mean difference, 3.0°; 95% CI, 1.4° to 4.6°; *p* < 0.001) in all angular definitions (Table 4). Anatomic inclination was significantly larger in females than in males (mean difference, 1.5°; 95% CI, 0.2° to 2.8°; *p* < 0.03).

**Table 3:** Difference between the pelvic coordinate system and the global system.

Group	Pelvic rotation	Pelvic tilt	Pelvic obliqueness
Male subjects (°)	3.09 (0.34 to 13.00)	1.94 (–12.65 to 12.36)	5.18 (0.90 to 12.86)
Female subjects (°)	3.61 (0.42 to 13.19)	3.35 (–9.23 to 19.21)	6.63 (0.48 to 23.37)
*p* values*	0.28	0.27	0.09
Overall (°)	3.35 (0.34 to 13.19)	2.64 (–12.65 to 19.21)	5.91 (0.48 to 23.37)

Data are presented as means with ranges.

* All *p* values were determined with the unpaired t-test.

**Table 4:** Comparison between males and females.

Group	Anatomic		Radiographic		Operative	
	Anteversion	Inclination	Anteversion	Inclination	Anteversion	Inclination
Male subjects (°)	18.8	52.8	14.8	51.2	22.9	48.7
	(9.1 to 31.0)	(45.6 to 60.1)	(7.3 to 25.0)	(41.8 to 57.6)	(10.9 to 36.5)	(38.7 to 55.6)
Female subjects (°)	21.5	54.3	17.3	52.2	26.9	48.6
	(5.9 to 33.1)	(47.8 to 60.5)	(4.5 to 26.8)	(45.9 to 58.4)	(7.0 to 39.2)	(41.3 to 57.0)
*p* values*	**0.029**	**0.029**	**0.0138**	0.141	**0.009**	0.856
Overall (°)	20.1	53.6	16.1	51.7	24.9	48.6
	(5.9 to 33.1)	(45.6 to 60.5)	(4.5 to 26.8)	(41.8 to 58.4)	(7.0 to 39.2)	(38.7 to 57.0)

Data are presented as means with ranges. Values indicating statistical significance are shown in **bold**.

* All *p* values were determined with the unpaired t-test.

**Fig 5:**
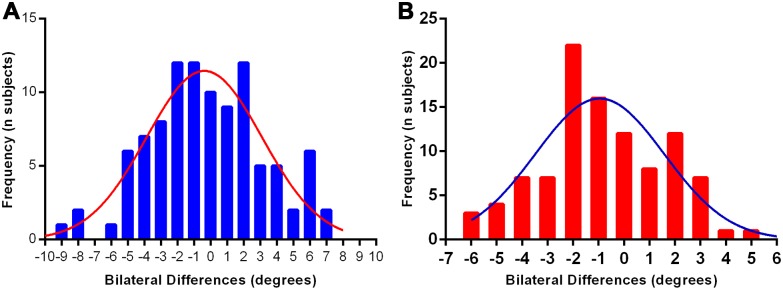
Frequency and magnitude of intrapatient bilateral differences (left minus right). **(A)** For anatomic anteversion, showing relative symmetry. **(B)** For anatomic inclination, showing relative symmetry.

Bilateral differences in anatomic anteversion were evenly distributed around a mean of 0.3° (maximum, 9.1°) (Fig 5A). Bilateral discrepancies in anatomic inclination were also evenly distributed, around a mean of 0.8° (maximum, 5.9°) (Fig 5B). However, a wide variation of acetabular orientation was observed among our subjects. For example, anatomic acetabular inclination ranged from 45.6° to 60.1° in males and from 47.8° to 60.5° in females, and anatomic anteversion ranged from 9.1° to 30.1° in males and from 5.9° to 33.1° in females (Table 4).

Compared with the data reported by Higgins et al. [4], acetabular anteversion and inclination were significantly smaller in our subjects (*p* < 0.001) for all angular definitions, except for operative anteversion and inclination (Table 5).

**Table 5:** Comparison between our study and Higgins et al.'s study.

Group	Anatomic		Radiographic		Operative	
	ΔAnteversion	ΔInclination	ΔAnteversion	ΔInclination	ΔAnteversion	ΔInclination
Male subjects	2.7°± 0.7°	2.9°±0.4°	2.7°± 0.6°	2.4°± 0.5°	1.1°± 0.9°	1.2°± 0.5°
	(**< 0.001)** *	**(< 0.001)**	**(< 0.001)**	**(< 0.001)**	(0.259)	(0.013)
Female subjects	3.2°± 0.8°	2.8°± 0.5°	5.7°± 0.6°	2.1°± 04°	5.7°± 1.1°	0.6°± 0.4°
	**(< 0.001)**	**(< 0.001)**	**(< 0.001)**	**(< 0.001)**	**(< 0.001**)	(0.198)
Overall	3.1°± 0.8°	2.9° ± 0.5°	3.1°± 0.7°	2.3°± 0.4°	5.7°± 1.0°	0.9°±0.4°
	(**< 0.001)**	**(< 0.001)**	**(< 0.001)**	**(< 0.001)**	**(< 0.001)**	(0.060)

Data are presented as means ± standard deviations. Significant differences are noted in **bold**

Δ All comparisons are Higgins’ data minus our data.

* All *p*-values are determined with unpaired t-test and presented in parentheses.

## Discussion

3D Acetabulometer is software developed in-house for virtual reconstruction of pelvic models from DICOM data. It uses a novel algorithm tailored to the segmentation of the hip joint. Semi-automatically measuring 3D acetabular orientation based on a reliable pelvic coordinate system was not only convenient, it was also statistically accurate and reliable. For patients with unilateral hip disease, measuring the contralateral normal hip joint with 3D Acetabulometer could provide individualized parameters for hip reconstruction or osteotomy; however, no reliable reference would be available for patients with bilateral hip disease or those with an asymmetrical pelvis, such as patients with developmental dysplasia of the hip with a high degree of deformity. The high accuracy of 3D Acetabulometer allowed the detection of tiny differences between the sexes, which provides supportive data for sex-specific reconstruction of the hip joint. The robust methodology makes it possible to perform 3D anatomic analyses on a large population, which potentially provides convincing information needed for sex-specific reconstruction regarding THA, periacetabular osteotomy, and correction of femoroacetabular impingement.

The original definitions of acetabular orientation reported by Murray were based on the global coronal, sagittal, and axial planes of the human body [1]. Variations in pelvic position affect the resulting angular measurement of the acetabular component or the native acetabulum [17, 18]. APP has been applied as a reliable reference, but it is a planar reference rather than a true 3D coordinate system. To establish a 3D coordinate system, the sagittal plane was previously defined to be normal to both the APP and a vector passing through bilateral ASISs residing in the APP [4]. This definition of the sagittal plane might not be the best choice, because the pelvis is not perfectly symmetrical in all subjects. In the current study, we have proposed a new way to determine the sagittal plane, which is to define the mirror plane associated with both ASIS regions, including a larger region of ilium, by using an iterative closest-point algorithm. We believe that semi-automatically choosing a larger ASIS region could compensate for the asymmetry of the pelvis, which could improve the reliability of measurement among patients with highly asymmetrical pelvises. In the current study, pelvic tilt, rotation, and obliqueness were all measured automatically on original CT images, and a large range of variation was observed (Table 3). Thus, 3D measurement of acetabular orientation is necessary to take pelvic posture into consideration. Indeed, our method was substantially more reliable than previously reported two-dimensional measures [8, 19].

Early studies regarding 3D measurement were restricted by the cost of the imaging, hardware, software, and skilled operators, and were therefore criticized for small sample sizes and lack of reliability analyses [10, 20]. With the development of imaging processing technology, the cost performance of 3D measurement has improved, which makes such studies more practical. Various computational algorithms have been reported for automatic detection of the acetabular rim [21, 22]. One study detected the acetabular rim using a level set algorithm that evolved on the surface of 3D pelvic models [21]; however, one of the eight mesh models failed to be segmented because it was over-smoothed by its algorithm. Other approaches lead to subjective errors during manual operations; these errors are hard to eliminate, which limits the clinical application of the approaches [19, 21, 22]. Another algorithm automatically selects points on the osseous ridge of the acetabulum and then generates a best-fit plane for describing acetabular orientation [4]. However, additional software is needed to finish the manual segmentation, which is a time-consuming process [4]. The in-house software used in the current study integrates segmentation, anatomical landmark identification, coordinate system establishment, and automatic acetabular orientation measurement, and it takes about 10 to 15 minutes to perform a complete analysis on a single subject. Thus, this technique can be efficiently applied in a larger population and may be helpful for providing a target for hip reconstruction in specific populations.

The 3D measurements obtained with 3D Acetabulometer indicated that female acetabula were significantly more anteverted than male acetabula in all angular definitions, similar to the findings of previous studies [4, 9, 11], whereas only the anatomic inclination of female acetabula was significantly bigger than that of male acetabula (*p* = 0.029). A higher baseline of acetabular inclination and anteversion may predispose females to more frequent and severe signs of dysplasia [14, 23]. Thanks to its higher precision, 3D Acetabulometer was able to detect small sex-specific differences in acetabular inclination, differences that were not statistically significant in a previous study [11].

Overall, both acetabular inclination and anteversion displayed a wide range of values in all angular definitions. For example, anatomic anteversion spanned 27.2° and anatomic inclination spanned 14.9°. The ranges of radiographic acetabular anteversion and inclination among the normal Chinese subjects in our study were partially outside the safe zone for THA proposed by Lewinnek [11, 24]. Further study is needed to clarify the significance of the safe zone for the guidance of cup placement.

Acetabular anteversion and inclination were smaller in our subjects than in a previously reported American population examined with a similar method [4] (Table 5). The demographics of that population were not reported, but it is likely that a minority of the subjects, if any, were of Asian descent. Therefore, the differences in our findings may be due to racial or ethnic differences in the study populations. However, further study is needed to demonstrate whether there is a racial difference in these parameters.

This study had limitations. First, 3D measurement based on computer modeling has been commonly criticized for oversimplification caused by surface modifications, including aggressive smoothing and surface simplification through mesh size reduction [9, 11, 12, 20, 21]. To address this, we selected high-resolution scans, and a smoothing algorithm with negligible change in volume (<0.1%) was included in 3D Acetabulometer. Second, our software has not been commercialized, so the reported technique cannot yet be used in the wider community. Third, we were interested in studying racial differences in acetabular anteversion and inclination, but the study we used for comparison [4] did not report detailed demographic information. A study specifically designed to address this issue will be needed.

A clinically available computer navigation system is able to achieve an accuracy of 3° regarding cup placement [25]. In the current study, we have shown that 3D Acetabulometer is perfectly reliable and achieves an accuracy of <0.5°. An accurate, reliable, and user-friendly software package has the potential to provide 3D anatomic parameters for sex-specific or even racial-specific reconstruction of the hip joint. Our result in a large sample of Chinese subjects provides solid data for guiding surgical reconstruction of hip joints in Chinese patients. Although 3D Acetabulometer is robust enough to detect small differences in acetabular orientation, further study is still needed to demonstrate its clinical significance.
